# Cardiovascular health and life expectancy with and without cardiovascular disease in the middle-aged and elderly Chinese population

**DOI:** 10.1186/s12889-023-17456-z

**Published:** 2023-12-18

**Authors:** Xue Xia, Shuohua Chen, Xue Tian, Qin Xu, Yijun Zhang, Xiaoli Zhang, Penglian Wang, Shouling Wu, Liming Lin, Anxin Wang

**Affiliations:** 1 Department of Neurology, Beijing Tiantan Hospital, Capital Medical University, Beijing, 100070 China; 2 China National Clinical Research Center for Neurological Diseases, Fengtai District, Beijing Tiantan Hospital, Capital Medical University. No, 119 South 4 Ring West Road, Beijing, 100070 China; 3 Department of Cardiology, Kailuan General Hospital. Tangshan 063000, Hebei, China; 4 Department of Epidemiology and Health Statistics, School of Public Health, Capital Medical University, Beijing, 100069 China; 5 Beijing Municipal Key Laboratory of Clinical Epidemiology, Beijing, 100069 China; 6 Cardiovascular Laboratory of Kailuan General Hospital. No, 57 Xinhua East Road, Tangshan, 063000 Hebei China

**Keywords:** Cardiovascular Health, Cardiovascular Disease, Life Expectancy

## Abstract

**Background:**

High cardiovascular health (CVH) was associated with lower risk of cardiovascular disease (CVD) and longer life expectancy. However, whether life years lived without CVD could increase faster than or at least at the same pace as total lifespan remains unknown. We aimed to explore the associations of CVH status with total life expectancy and life years lived with and without CVD among middle-aged and elderly men and women.

**Methods:**

We included 65,587 participants aged ≥ 45 years from Kailuan study, who were recruited during June 2006 to October 2007. CVH was scored and classified (low [0–49 points], moderate [50–79 points] and high [80–100 points]) with *Life’s Essential 8*, incorporating evaluations of health behaviors and factors. All-cause mortality and incident non-fatal CVD were recorded from baseline to December 31, 2020. The multi-state life table was adopted to explore the associations of CVH status with total life expectancy and life years lived with and without CVD.

**Results:**

Six thousand fifty eight cases of incident non-fatal CVD and 10,580 cases of deaths were identified. Men aged 45 years with low, moderate, and high CVH had a life expectancy of 33.0, 36.5 and 38.5 years, of which 7.8 (23.6%), 6.0 (16.3%) and 3.7 years (9.6%) were spent with CVD. For women, the corresponding life expectancy was 36.6, 43.6 and 48.6 years, and the remaining life years lived with CVD were 7.8 (21.3%), 6.0 (13.7%) and 4.5 years (9.3%), respectively. The benefits of high CVH were persistent across lifespan from age 45 to 85 years and consistent when CVH was evaluated with health behaviors and factors alone.

**Conclusions:**

High CVH compared with low CVH was associated with longer total life expectancy and fewer years spent with CVD, indicating that promoting CVH is of great importance for CVD prevention and healthy ageing in China.

**Supplementary Information:**

The online version contains supplementary material available at 10.1186/s12889-023-17456-z.

## Background

Aging, an inevitable part of life, has acted as one of the most critical determinants of cardiovascular health [1]. Despite dramatic improvements in health care and public health education, individuals remain at higher lifetime risk of cardiovascular disease (CVD) in the process of ageing worldwide [2]. In China, the prevalence of CVD have substantially increased by 99.8% since 1990 along with longer life expectancy, which led to approximately 10.4 million years lived with disability (YLDs) in 2019 [3].

A series of modifiable cardiovascular risk factors were identified through decades of epidemiological studies, and adherence to a combination of health behaviors and health factors has been proven to be an effective approach to prevent the occurrence of CVD and improve the prognosis [4–6]. Accordingly, the American Heart Association (AHA) proposed a comprehensive concept of ideal cardiovascular health (CVH) called *Life Simple 7* in 2010, and recently proposed its updated version namely *Life’s Essential 8* (LE8), which was enhanced and expanded to better assess CVH of individuals [7, 8]. Moreover, in addition to attenuating CVD risks, population-based evidence has also indicated that lifestyle intervention was associated with decreased all-cause mortality and increased life expectancy [9, 10].

Consequently, it might be plausible that maintaining ideal CVH could delay CVD to later ages and help individuals with worse health survive longer than they would have in the past, while the overall prevalence and years lived with CVD may not actually alter or even increase. To better translate observed benefits of CVH modifications to public health interventions, it is of great importance to gain more insight into the impact of overall lifestyle intervention on both incident CVD and total life expectancy, with special attention paid to the proportions of remaining years lived with CVD under an expanded lifespan.

Therefore, we conducted a multi-state life table (MSLT) analysis based on the Kailuan cohort involving adults from Northern China to explore the associations of CVH status defined by the new LE8 metrics with total life expectancy and years lived with and without CVD for the middle-aged and elderly men and women independently, aiming to provide evidence for primordial CVD prevention and healthy ageing in China.

## Methods

### Study population

The Kailuan study is a prospective cohort study based on the Kailuan community in Tangshan city, Northern China. Detailed designs of the study were reported in the previous publications [11–13]. In brief, the recruitment process was initiated by the Kailuan company in 2006, inviting community population aged ≥ 18 years to participated in the baseline survey. During June 2006 to October 2007, a total of 101,510 adults, including 81,110 men and 20,400 women, participated in the baseline survey, and were subsequently followed up each two years until their deaths or the most recent visit finished on December 31, 2020, whichever occurred first. In accordance with the World Health Organization (WHO) definition of the middle-aged and elderly population, we firstly excluded 29,635 participants aged less than 45 years at baseline, and further excluded 6,288 participants with missing data on baseline LE8 metrics. In total, 65,587 participants aged ≥ 45 years were ultimately included in the current analyses (Supplementary Fig. 1). The comparison of baseline characteristics between included and excluded individuals is presented in Supplementary Table 1.

### Data collection

Information on demographic characteristics, lifestyle information, history of present illness, and medication usage was collected at baseline and during each biennial follow-up visit with uniformed questionnaires. Habitual dietary intake was collected through asking the frequency of consuming typical food items during the past year. Self-reported time spent on both physical activity and sleep was recorded, as well as the current and former use of cigarettes. In addition, social and economic status, represented by educational levels, household annual income per capita and marital status, was also investigated. Fasting blood samples were drawn for measurements of serum glucose and lipid concentrations. Physical examinations were conducted by trained and certificated local staff. Weights and heights were measured with lightweight clothing and no shoes. Blood pressure measurements were obtained using mercury sphygmomanometers, and the mean of three readings was used in the analyses.

### CVH definition

CVH of each participant was assessed with the recently updated LE8 metrics, which included evaluation of both health behavior domain (diet, physical activity, tobacco/nicotine exposure, and sleep health) and health factor domain (body mass index [BMI], blood lipids, blood glucose, and blood pressure). Detailed definitions and scorings for all eight metrics of CVH were presented in Supplementary Table 2. In short, the score of each metric ranged from the lowest of 0 points to the highest of 100 points, and the overall and domain-specific CVH were accordingly scored as the unweighted average of all included metrics, with the higher score indicating a better CVH. In line with recommendations from the AHA, we further defined CVH status with 80–100 points as high CVH, 50–79 points as moderate CVH, and 0–49 points as low CVH, respectively.

### Health states

CVD was defined as a composite of myocardial infarction (MI), atrial fibrillation, heart failure and stroke. More specifically, myocardial infarction was diagnosed based on clinical symptoms, electrocardiography and dynamic changes in levels of cardiac enzyme and other biomarkers [14]. Atrial fibrillation was clinically identified with absolutely irregular RR intervals and no discernible, distinct *P* values [15]. Diagnosis of heart failure was confirmed according to clinical symptoms, chest radiography, echocardiography, and electrocardiography [16]. Stroke was diagnosed on the basis of neurological signs, clinical symptoms, and neuroimages from computed tomography or magnetic resonance imaging scans [17].

In the current study, health states of participants were classified into three categories (free of CVD, living with CVD, and death), which were dynamically updated during follow-ups from the baseline survey till their deaths or the latest visit. As previously reported, the vital status of participants was identified based on the death certificates from provincial vital statistics offices, and the physician-diagnosed CVD was mainly confirmed through the biennial face-to face interviews [18]. As complements, medical records from the Municipal Social Insurance Institution covering the entire Kailuan cohort and discharge summaries from eleven hospitals in the Kailuan community were further gathered and cross-validated, and all potential cases were reviewed and ascertained by the central expert panel who were unaware of the study design.

### Statistical analysis

All analyses were conducted separately for men and women. Baseline characteristics were presented as mean (standard deviation [SD]) or frequency (%), as appropriate. The MSLT was adopted to evaluate the associations of CVH status with the total life expectancy and the number of years that participants could expect to live with and without CVD, respectively. Detailed descriptions of MSLT have been presented previously [19, 20]. In simple terms, the MSLT is a time-inhomogeneous, finite-space, continuous-time Markov model, which characterizes population movement over time in a finite, discrete and mutually exclusive state space as a Markov process. Three health states (free of CVD, living with CVD, and death) were defined, and we thus assumed three possible transitions included from free of CVD to living with CVD, from free of CVD to death, and from living with CVD to death. Backflows were not allowed (e.g., from CVD to free of CVD) and only the first event into a state was considered. The age-specific state-dependent transition probabilities was firstly estimated with multinomial logistic regression, adjusted by educational levels, household annual income per capita and marital status [21]. In the analysis of CVH defined with health behaviors alone, health factors were further adjusted and vice versa. Then, with the microsimulation approach, we simulated a cohort of 100 000 people aged 45 years and fitted the survival trajectory of the cohort population with transition probability between different health states calculated based on the MSLT, thus estimating the survival time in different health states of each individual in the cohort from 45 years to death. Ultimately, the mean values of overall survival time as well as the average time living with or free from CVD were calculated as life expectancy and years lived with/without CVD.

To further evaluate the robustness of our findings, sensitivity analyses were conducted by 1) involving time-varying CVH status of LE8 metrics at each visit instead of the baseline CVH status alone; 2) excluding non-fatal cardiovascular events and deaths occurred in the first year. All analyses were performed with SAS version 9.4 (SAS Institute Inc., Cary, NC, USA). The SPACE program was adopted to estimate MSLT functions via microsimulation [22]. Tests were two-sided, with the statistical significance set at *P* < 0.05.

## Results

Of all the 65,587 participants included, 53,775 (82.0%) were men and 11,812 (18.0%) were women. With categorical considerations suggested by the AHA, 6,628 (12.3%) of the men were classified into the low CVH status, 44,243 (82.3%) into the moderate CVH status, and 2,904 (5.4%) into the high CVH status. By comparison, the CVH status of women was generally better; the corresponding number (%) of each category was 388(3.3%), 9,754(82.6%) and 1,670(14.1%), respectively. Participants with more advantageous CVH scores were older in men while younger in women. In addition, they were less likely to have a history of CVD before baseline, and more likely to be married and of higher socioeconomic status, regardless of gender (Table 1).

**Table 1:** Baseline characteristics of women and men in Kailuan population by CVH Status of LE8

	**Men**			**Women**		
	**low CVH**	**moderate CVH**	**high CVH**	**low CVH**	**moderate CVH**	**high CVH**
**Participants, N (%)**	6628(12.3)	44,243(82.3)	2904(5.4)	388(3.3)	9754(82.6)	1670(14.1)
**Demographics**						
Age at baseline, Mean (SD), in years	55.6(7.9)	57.6(8.9)	59.0(9.5)	58.3(7.8)	55.4(7.7)	52.9(6.8)
High school or above, N (%)	841(12.7)	5023(11.4)	411(14.2)	53(13.7)	1893(19.4)	470(28.1)
Personal monthly income ≥ 600 CNY, N (%)	3681(55.5)	31,229(70.6)	2233(76.9)	298(76.8)	7972(81.7)	1367(81.9)
Married, N (%)	6240(94.1)	42,347(95.7)	2768(95.3)	342(88.1)	9099(93.3)	1569(94.0)
**CVH scores of LE8, Mean (SD), out of 100 possible points**						
Overall CVH	43.9(5.2)	64.9(7.7)	83.4(2.8)	45.5(3.8)	68.0(7.4)	83.8(2.9)
Health behaviors	39.8(13.0)	60.9(13.5)	75.2(7.8)	55.3(13.3)	68.9(8.6)	76.0(7.7)
Diet	34.9(17.4)	38.9(15.3)	43.7(17.5)	33.5(15.1)	36.8(13.0)	43.2(16.5)
Physical activity	41.1(30.5)	57.1(24.5)	67.6(24.4)	44.8(27.1)	56.1(22.1)	67.4(24.0)
Tobacco/nicotine exposure	14.8(30.9)	60.0(46.2)	94.5(20.0)	81.4(37.2)	97.7(14.1)	99.7(4.6)
Sleep health	68.6(29.7)	87.4(21.7)	95.1(13.2)	61.4(32.0)	85.0(24.3)	93.6(14.4)
Health factors	48.0(13.3)	69.0(14.4)	91.6(7.2)	35.8(12.6)	67.0(14.7)	91.5(7.3)
BMI	53.0(20.9)	67.8(23.3)	91.9(15.5)	41.0(20.7)	63.3(24.4)	89.8(16.5)
Blood lipids (non-HDL cholesterol)	49.4(28.0)	73.6(27.7)	93.6(15.6)	38.0(26.9)	67.9(29.2)	90.5(18.8)
Blood glucose	63.5(30.1)	84.5(24.4)	97.4(10.6)	47.4(32.8)	84.7(25.4)	98.4(8.2)
BP	25.9(30.0)	50.0(33.4)	83.7(18.4)	16.7(26.0)	52.3(33.3)	87.4(17.0)
**Baseline history of CVD, N (%)**	641(9.7)	2381(5.4)	86(3.0)	47(12.1)	346(3.5)	28(1.7)

*Abbreviations*: *BMI* Body mass index, *BP* Blood pressure, *CNY* Chinese yuan, *CVD* Cardiovascular disease, *CVH* Cardiovascular health, *HDL* High-density lipoprotein, *LE8* Life’s Essential 8; SD, standard deviation

Values were represented by mean (SD) for continuous variables and frequency (%) for categorical variables

**Fig. 1:**
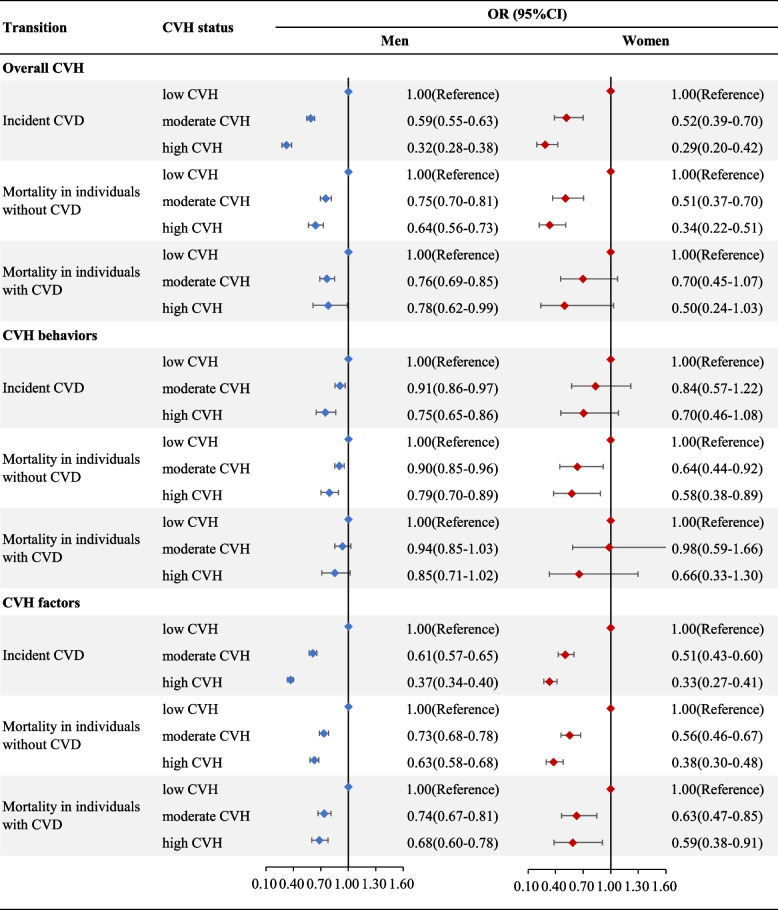
Associations between CVH Status of LE8 and risk of incident CVD and all-cause mortality in men and women. *Abbreviations*: *CVD* Cardiovascular disease, *CVH* cardiovascular health, *LE8* Life’s Essential 8, *OR (95%CI)* Odds ratio (95% confidence interval)

Figure 1 presents the association between CVH status of LE8 and risk of incident CVD and all-cause mortality in men and women, and the component-specific results were listed in the Supplementary Fig. 2. During follow-ups from the baseline survey to December 31, 2020, 6,058 cases of incident non-fatal CVD and 10,580 cases of deaths were recorded. High CVH was associated with a significantly decreased risk of incident CVD in both men (odds ratio [OR] 0.32, 95% confidence interval [95%CI] 0.28–0.38) and women (OR 0.29, 95%CI 0.20–0.42), in comparison with those with low CVH. More specifically, the OR (95%CI) for incident CVD of high CVH defined by health behaviors was 0.75(0.65–0.86) for men and 0.70(0.46–1.08) for women, and the corresponding OR(%CI) of CVH status defined by health factors was 0.37 (0.34–0.40) and 0.33(0.27–0.41), respectively. In addition, similar tendencies were also observed for the risk of all-cause mortality, for individuals both living with and without CVD.

**Fig. 2:**
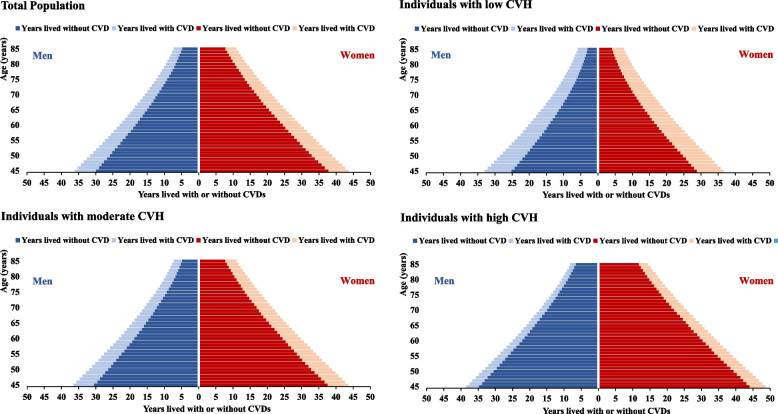
Life expectancy and years lived with or without CVD at a given age from 45 to 85 years by CVH Status of LE8 in men and women. *Abbreviations*: *CVD* Cardiovascular disease, *CVH* Cardiovascular health, *LE8* Life’s Essential 8

The total life expectancy and years lived with and without CVD in men and women are illustrated in Table 2 and Fig. 2. In general, life expectancy at age 45 years was 36.1 years for men, of which 6.2 years, corresponding to 17.2% of total life expectancy, were lived with CVD. For women, the total life expectancy was 43.6 years and 5.8 years (13.4%) were lived with CVD. Compared with individuals with low CVH, remarkable longer total life expectancy and shortened years lived with CVD were observed for those with moderate and high CVH. Life expectancy at age 45 years of low, moderate, and high CVH status was 33.0 years, 36.5 years and 38.5 years in men and 36.6 years, 43.6 years, and 48.6 years in women, respectively. By contrast, the corresponding years lived with CVD, at age 45 years, decreased from 7.8 years to 3.7 years in men and from 7.8 years to 4.5 years in women. When further classified by initial health states, high CVH was related to both improved life expectancy and declined years lived with CVD among individuals without CVD; while for those with CVD, high CVH was found to be associated with longer life years spent with CVD (Supplementary Table 3).

**Table 2:** Life expectancy and years lived with or without CVD at age 45 years by CVH Status of LE8

CVH status	Men			Women		
	**Life expectancy, years**	**Years lived without CVD, years (%)**	**Years lived with CVD, years (%)**	**Life expectancy, years**	**Years lived without CVD, years (%)**	**Years lived with CVD, years (%)**
**Overall CVH**						
low CVH	33.0	25.2(76.4)	7.8(23.6)	36.6	28.8(78.7)	7.8(21.3)
moderate CVH	36.5	30.6(83.7)	6.0(16.3)	43.6	37.6(86.3)	6.0(13.7)
high CVH	38.5	34.8(90.4)	3.7(9.6)	48.6	44.1(90.7)	4.5(9.3)
**Health behaviors**						
low CVH	35.3	28.9(81.8)	6.4(18.2)	40.2	34.0(84.6)	6.2(15.4)
moderate CVH	36.3	30.3(83.5)	6.0(16.5)	44.0	38.4(87.2)	5.6(12.8)
high CVH	37.8	32.2(85.3)	5.5(14.7)	46.0	39.9(86.6)	6.2(13.4)
**Health factors**						
low CVH	32.6	24.8(76.0)	7.8(24.0)	37.6	29.8(79.4)	7.7(20.6)
moderate CVH	36.3	29.8(82.1)	6.5(17.9)	44.3	38.2(86.4)	6.0(13.6)
high CVH	38.2	33.8(88.3)	4.5(11.7)	48.2	43.4(90.2)	4.7(9.8)
**Total population**	36.1	29.9(82.8)	6.2(17.2)	43.6	37.8(86.6)	5.8(13.4)

*Abbreviations*: *CVD* Cardiovascular disease, *CVH* Cardiovascular health, *LE8* Life’s Essential 8

**Fig. 3:**
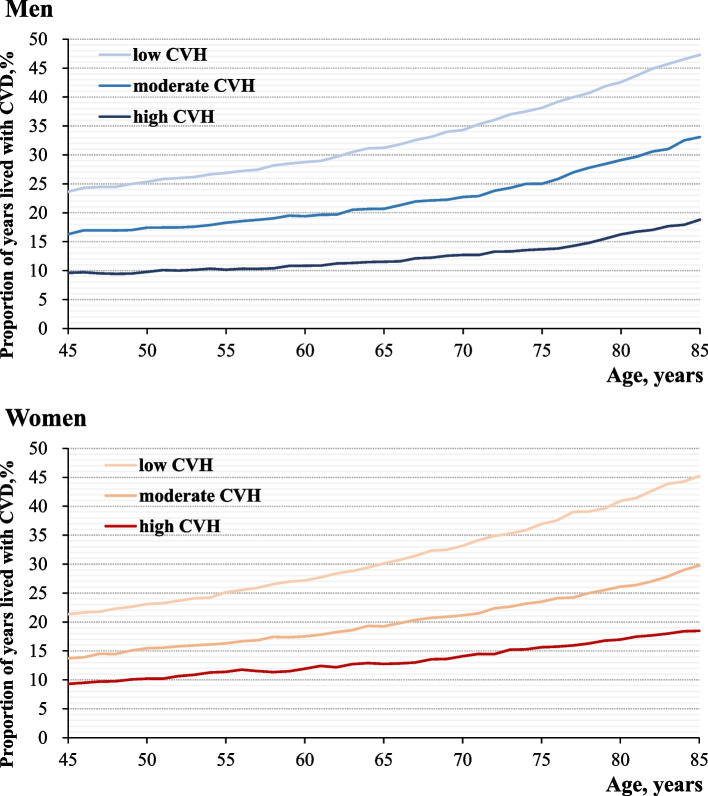
Proportion of years lived with CVD at a given age from 45 to 85 years by CVH Status of LE8 in men and women. Proportion is computed by dividing years lived with CVD by total life expectancy at a given age from 45 to 85 years. *Abbreviations*: *CVD* Cardiovascular disease, *CVH* Cardiovascular health, *LE8* Life’s Essential 8

Moreover, the benefits of high CVH were observed to be persistent across the lifespan (Fig. 3, Supplementary Table 4, Supplementary Table 5). For instance, women with low, moderate, and high CVH at age 45 years spent 21.3%,13.7% and 9.3% of their total life expectancy with CVD, and the corresponding proportions of years lived with CVD at age 85 years were 45.2%, 29.8%, and 18.5%, respectively. Besides, as was shown in Supplementary Table 6–7, the results of sensitivity analyses were generally consistent with primary findings.

## Discussion

This prospective population-based cohort study indicated that in comparison with participants with low CVH, maintaining high CVH was associated with longer total life expectancy and fewer years spent with CVD across the lifespan, which contributed to a larger proportion of CVD-free life years. It was estimated that the total life expectancy at age 45 years for men and women with low CVH in the Kailuan cohort was 33.0 and 36.6 years, of which 23.6% and 21.3% were spent with CVD. For those with moderate and high CVH, lifespan was expanded (36.5 years and 38.5 years for men; 43.6 years and 48.6 years for women) and less of their remaining life expectancy (16.3% and 9.6% for men; 13.7% and 9.3% for women) was spent with CVD. Considering the less-than-ideal CVH status of the middle-aged and elderly Chinese, [23] our study had a strong practical implication for CVD prevention and healthy ageing in China, providing basis for estimating future healthcare costs and planning for healthcare needs.

High CVH, including adherence to healthy lifestyles and maintenance of ideal cardio-metabolic factors, has long been proved to be associated with decreased risk of incident CVD, which significantly delays the onset of CVD and improves the survival of CVD patients [4, 6, 24, 25]. Moreover, it is also recognized as a strong predictor for extended life expectancy [10, 26]. However, since individuals turn to be more vulnerable to CVD once they reach older ages, it was confusing that whether the benefits of maintaining high CVH could be fully translated into healthy longevity. The present multi-state life expectancy analysis, which considered both CVD morbidity and all-cause mortality, provided further estimates on years spent with and without CVD, respectively. For individuals free from CVD, decreased risks of CVD and mortality were observed to be associated with high CVH, which indicated that the occurrence of CVD could be postponed in older ages and the lifespan could be expanded, together contributing to increased life years lived free from CVD. On the other hand, a decreased risk of mortality was observed for CVD patients with high CVH. In other words, after the occurrence of CVD, more remaining years were expected to be lived if they maintained a high CVH. Furthermore, the benefits of high CVH were persistent across the lifespan from age 45 to 85 years, suggesting that achieving a better CVH status is of great importance for preventing CVD events and pursuing healthy longevity, even for those aged population.

Although a series of studies have investigated the association of CVH with risk of CVD and all-cause mortality separately, evidence on life expectancy with and without CVD according to different CVH status, to the best of our knowledge, is still lacking. Most previous publications focused on the relative risk of incident CVD or the remaining life years free of CVD. The project of the Prediction for Atherosclerotic Cardiovascular Disease Risk in China, for instance, has reported a gradient inverse association between the number of ideal CVH metrics assessed with the origin LS7 metrics and the hazard ratio of CVD [23]. A previous analysis based on the Kailuan Study, also adopting the LS7 metrics, indicated that life years free from CVD at age 35 years were 38.4, 45.0, and 50.3 years in participants with consistently low, moderate, and high CVH, respectively [27]. Additionally, several studies further examined the impact of combined lifestyle risk factors on the total life expectancy. The Singapore Chinese Health Study observed that adherence to 4–5 healthy lifestyle factors versus none could achieve a gain of 8.1 years in women and 6.6 years in men for the life expectancy at age 50 years [26]. Another study conducted in the U.S. indicated that adherence to 5 low-risk lifestyle factors could prolong life expectancy at age 50 years by 12.2 and 14.0 years for male and female adults, respectively [10]. More relevant, the Rotterdam Study, investigating the effects of lifestyle on life expectancy with and without heart failure, concluded that a healthy overall lifestyle was associated with a longer total life expectancy and years lived without heart failure [20]. Our findings were generally consistent with the above-mentioned studies, and we further provided more comprehensive evidence that a high CVH may improve not only total life expectancy but also life years without CVD.

Even though health behaviors had a strong influence on the major CVD risk factors, we still found significant benefits of ideal health behaviors under adjustment for CVH defined by health factors. These findings indicated that the protection afforded by health behaviors might involve mechanisms beyond merely optimizing levels of cardio-metabolic factors, which further emphasized the crucial roles of health behaviors for CVD prevention and healthy ageing. Moreover, as for different genders, we observed that women lived longer in general and could gain more total life expectancy through keeping a high CVH than men. The improvements in the proportion of CVD-free life years, however, were similar in men and women. This difference supported the hypothesis that the preventive effect of high CVH on incident CVD might be comparable across genders, while its protection from total deaths seemed to be more remarkable in women. Nevertheless, more research is warranted to further clarify the mechanism of gender differences in the future.

This study has several strengths. First, previous studies more commonly evaluated the association of CVH with life expectancy for individual with or without CVD respectively, [28] or used relatively indirect methods like competing risk adjusted Cox proportional hazards functions or penalized spline model combined with Irwin restricted mean [29, 30]. With the use of MSLT, the influences of high CVH on both morbidity (incident CVD) and mortality (life expectancy) were simultaneously considered in the current analysis, which provided a more intuitive and comprehensive estimates of potential benefits from maintaining ideal CVH status. Secondly, most existing studies investigated the impact of lifestyle factors on life expectancy at an old age [31]. Our current study expanded previous findings to support benefits of starting a healthy lifestyle early at a younger age and further explored the association between CVH status and CVD incidence and mortality across different age stages. Thirdly, our study adopted the latest LE8 definition of CVH, which was more comprehensive than previous studies, considering both health behaviors and health factors [9]. And we modified some definitions of LE8 components to obtain conclusions more suitable for the Chinese population. For instance, we used the Chinese criteria of BMI with lower cutting-off values instead of the WHO criteria, since Asians generally had a higher percentage of body fat than white people of the same age, sex, and BMI. Other strengths included the prospective cohort design, the large sample size, and the long-term follow-up.

However, there were also several limitations to be acknowledged. Firstly, information on lifestyle like diet and physical activity was self-reported, which may potentially suffer from misclassification and residual confounding, leading to imprecise estimates. Given the consistency of our primary results with time-varying sensitivity analysis and previous publications, we believe that our findings were valid and reliable in general. Secondly, other crucial CVD risk factors like psychological status and genetic predisposition were not evaluated due to lack of data. More evidence on the potential effects of psychological health as well as genetic susceptibility is urgently needed. Additionally, given the fact that the Kailuan study only reflected the incidence and mortality of specific cohort population over a certain follow-up period, the absolute value of life-years estimated in our study might not be a representative statistic for the current China. Nevertheless, the potential benefits of high CVH on life expectancy and years lived with/without CVD observed in the current analysis could still be generalized to other population, since the association between CVH and life expectancy were less likely to be distorted by the distributions of CVH risk factors.

## Conclusions

The present study indicated that maintaining a high CVH was related to not only longer total life expectancy but also more remaining life years lived free of CVD in the middle-aged and elderly men and women, which provided support for promoting CVH as an important strategy for CVD prevention and healthy ageing in China.

### Supplementary Information

**Additional file 1: Supplementary Table 1.** Baseline characteristics between excluded and included participants.** Supplementary Table 2.** Definition and scoring approach for quantifying CVH of LE8. **Supplementary Table 3. **Life expectancy and years lived with or without CVD at age 45 years by CVH Status of LE8, according to initial states of CVD. **Supplementary Table 4. **Life expectancy and years lived with CVD at a given age from 45 years to 85 years by CVH Status of LE8 in men. **Supplementary Table 5. **Life expectancy and years lived with CVD at a given age from 45 years to 85 years by CVH Status of LE8 in women. **Supplementary Table 6.** Life expectancy and years lived with or without CVD at age 45 years by time-varying CVH Status of LE8. **Supplementary Table 7. **Life expectancy and years lived with or without CVD at age 45 years by CVH Status of LE, excluding non-fatal cardiovascular events and deaths occurred in the first year. **Supplementary Table 8. **Life expectancy and years lived with or without CVD at age 45 years by CVH Status of LE, excluding non-fatal cardiovascular events and deaths occurred in the first year. **Supplementary Figure 1. **Flow chart of participants included and excluded in the analyses. **Supplementary Figure 2. **Associations between CVH Status of component-specific LE8 and risk of incident CVD and all-cause mortality in men and women. Abbreviations: CVD, cardiovascular disease; CVH, cardiovascular health; LE8, Life’s Essential 8; OR (95%CI), odds ratio (95% confidence interval).

## Data Availability

Data may be available from the corresponding author on a reasonable request.

## Abbreviations

CVH: Cardiovascular health
CVD: Cardiovascular disease
AHA: American Heart Association
BMI: Body mass index
LE8: Life’s Essential 8
MSLT: Multi-state life table
SD: Standard deviation
WHO: World Health Organization
YLDs: Years lived with disability

## References

[1] North BJ, Sinclair DA (2012). The intersection between aging and cardiovascular disease. Circ Res.

[2] Roth GA, Mensah GA, Johnson CO, Addolorato G, Ammirati E, Baddour LM (2020). Global Burden of Cardiovascular Diseases and Risk Factors, 1990–2019: Update From the GBD 2019 Study. J Am Coll Cardiol.

[3] Institute for Health Metrics and Evaluation, Global Burden of Disease Study 2019 (GBD 2019) Data Resources: IHME. 2019. [Available from: https://vizhub.healthdata.org/gbd-compare/.

[4] Dong C, Rundek T, Wright CB, Anwar Z, Elkind MS, Sacco RL (2012). Ideal cardiovascular health predicts lower risks of myocardial infarction, stroke, and vascular death across whites, blacks, and hispanics: the northern Manhattan study. Circulation.

[5] Zhang Q, Zhou Y, Gao X, Wang C, Zhang S, Wang A (2013). Ideal cardiovascular health metrics and the risks of ischemic and intracerebral hemorrhagic stroke. Stroke.

[6] Lv J, Yu C, Guo Y, Bian Z, Yang L, Chen Y (2017). Adherence to Healthy Lifestyle and Cardiovascular Diseases in the Chinese Population. J Am Coll Cardiol.

[7] Lloyd-Jones DM, Hong Y, Labarthe D, Mozaffarian D, Appel LJ, Van Horn L (2010). Defining and setting national goals for cardiovascular health promotion and disease reduction: the American Heart Association's strategic Impact Goal through 2020 and beyond. Circulation.

[8] Lloyd-Jones DM, Allen NB, Anderson CAM, Black T, Brewer LC, Foraker RE (2022). Life’s Essential 8: Updating and Enhancing the American Heart Association’s Construct of Cardiovascular Health: A Presidential Advisory From the American Heart Association. Circulation..

[9] Sun Q, Yu D, Fan J, Yu C, Guo Y, Pei P (2022). Healthy lifestyle and life expectancy at age 30 years in the Chinese population: an observational study. Lancet Public Health..

[10] Li Y, Pan A, Wang DD, Liu X, Dhana K, Franco OH (2018). Impact of Healthy Lifestyle Factors on Life Expectancies in the US Population. Circulation.

[11] Wu S, An S, Li W, Lichtenstein AH, Gao J, Kris-Etherton PM (2019). Association of Trajectory of Cardiovascular Health Score and Incident Cardiovascular Disease. JAMA Netw Open.

[12] Wang C, Yuan Y, Zheng M, Pan A, Wang M, Zhao M (2020). Association of Age of Onset of Hypertension With Cardiovascular Diseases and Mortality. J Am Coll Cardiol.

[13] Zhou YF, Chen S, Wang G, Chen S, Zhang YB, Chen JX (2022). Effectiveness of a Workplace-Based, Multicomponent Hypertension Management Program in Real-World Practice: A Propensity-Matched Analysis. Hypertension.

[14] Tunstall-Pedoe H, Kuulasmaa K, Amouyel P, Arveiler D, Rajakangas AM, Pajak A (1994). Myocardial infarction and coronary deaths in the World Health Organization MONICA Project. Registration procedures, event rates, and case-fatality rates in 38 populations from 21 countries in four continents. Circulation.

[15] Kirchhof P, Benussi S, Kotecha D, Ahlsson A, Atar D, Casadei B (2016). 2016 ESC Guidelines for the management of atrial fibrillation developed in collaboration with EACTS. Eur Heart J.

[16] Swedberg K, Cleland J, Dargie H, Drexler H, Follath F, Komajda M (2005). Guidelines for the diagnosis and treatment of chronic heart failure: executive summary (update 2005): The Task Force for the Diagnosis and Treatment of Chronic Heart Failure of the European Society of Cardiology. Eur Heart J.

[17] The World Health Organization. Special Report from the World Health Organization. Stroke-1989 Recommendations on stroke prevention, diagnosis, and therapy. Report of the WHO Task Force on Stroke and other Cerebrovascular Disorders. Stroke. 1989;20(10):1407–31. 10.1161/01.str.20.10.1407 2799873

[18] Wang YH, Wang J, Chen SH, Li JQ, Lu QD, Vitiello MV (2020). Association of Longitudinal Patterns of Habitual Sleep Duration With Risk of Cardiovascular Events and All-Cause Mortality. JAMA Netw Open.

[19] Dhana K, Franco OH, Ritz EM, Ford CN, Desai P, Krueger KR, et al. Healthy lifestyle and life expectancy with and without Alzheimer’s dementia: population based cohort study. BMJ. 2022;377:e068390. 10.1136/bmj-2021-068390 PMC9006322 35418416

[20] Limpens MAM, Asllanaj E, Dommershuijsen LJ, Boersma E, Ikram MA, Kavousi M (2022). Healthy lifestyle in older adults and life expectancy with and without heart failure. Eur J Epidemiol.

[21] Laditka SB, Wolf DA (1998). New Methods for Analyzing Active Life Expectancy. J Aging Health.

[22] Cai L, Hayward MD, Saito Y, Lubitz J, Hagedorn A, Crimmins E (2010). Estimation of multi-state life table functions and their variability from complex survey data using the SPACE Program. Demogr Res.

[23] Han C, Liu F, Yang X, Chen J, Li J, Cao J (2018). Ideal cardiovascular health and incidence of atherosclerotic cardiovascular disease among Chinese adults: the China-PAR project. Sci China Life Sci.

[24] Han Y, Hu Y, Yu C, Guo Y, Pei P, Yang L (2021). Lifestyle, cardiometabolic disease, and multimorbidity in a prospective Chinese study. Eur Heart J.

[25] Vasan RS, Enserro DM, Xanthakis V, Beiser AS, Seshadri S (2022). Temporal Trends in the Remaining Lifetime Risk of Cardiovascular Disease Among Middle-Aged Adults Across 6 Decades: The Framingham Study. Circulation.

[26] Pan XF, Li Y, Franco OH, Yuan JM, Pan A, Koh WP (2020). Impact of Combined Lifestyle Factors on All-Cause and Cause-Specific Mortality and Life Expectancy in Chinese: The Singapore Chinese Health Study. J Gerontol A Biol Sci Med Sci.

[27] Wang L, Song L, Li D, Zhou Z, Chen S, Yang Y (2021). Ideal Cardiovascular Health Metric and Its Change With Lifetime Risk of Cardiovascular Diseases: A Prospective Cohort Study. J Am Heart Assoc.

[28] Xu C, Zhang P, Cao Z (2022). Cardiovascular health and healthy longevity in people with and without cardiometabolic disease: A prospective cohort study. EClinicalMedicine.

[29] Allen NB, Zhao L, Liu L, Daviglus M, Liu K, Fries J (2017). Favorable Cardiovascular Health, Compression of Morbidity, and Healthcare Costs: Forty-Year Follow-Up of the CHA Study (Chicago Heart Association Detection Project in Industry). Circulation.

[30] Berkelmans GFN, Gudbjörnsdottir S, Visseren FLJ, Wild SH, Franzen S, Chalmers J (2019). Prediction of individual life-years gained without cardiovascular events from lipid, blood pressure, glucose, and aspirin treatment based on data of more than 500 000 patients with Type 2 diabetes mellitus. Eur Heart J.

[31] Wang J, Chen C, Zhou J, Ye L, Li Y, Xu L (2023). Healthy lifestyle in late-life, longevity genes, and life expectancy among older adults: a 20-year, population-based, prospective cohort study. Lancet Healthy Longev.

