# Supplementary material for: Cardiovascular health and life expectancy with and without cardiovascular disease in the middle-aged and elderly Chinese population
Source: BMC Public Health. 2023 Dec 18;23:2528. doi: 10.1186/s12889-023-17456-z (PMC10726610; doi:10.1186/s12889-023-17456-z)
Supplement: Supplementary file 1 — Additional file 1: Supplementary Table 1. Baseline characteristics between excluded and included participants. Supplementary Table 2. Definition and scoring approach for quantifying CVH of LE8. Supplementary Table 3. Life expectancy and years lived with or without CVD at age 45 years by CVH Status of LE8, according to initial states of CVD. Supplementary Table 4. Life expectancy and years lived with CVD at a given age from 45 years to 85 years by CVH Status of LE8 in men. Supplementary Table 5. Life expectancy and years lived with CVD at a given age from 45 years to 85 years by CVH Status of LE8 in women. Supplementary Table 6. Life expectancy and years lived with or without CVD at age 45 years by time-varying CVH Status of LE8. Supplementary Table 7. Life expectancy and years lived with or without CVD at age 45 years by CVH Status of LE, excluding non-fatal cardiovascular events and deaths occurred in the first year. Supplementary Table 8. Life expectancy and years lived with or without CVD at age 45 years by CVH Status of LE, excluding non-fatal cardiovascular events and deaths occurred in the first year. Supplementary Figure 1. Flow chart of participants included and excluded in the analyses. Supplementary Figure 2. Associations between CVH Status of component-specific LE8 and risk of incident CVD and all-cause mortality in men and women. Abbreviations: CVD, cardiovascular disease; CVH, cardiovascular health; LE8, Life’s Essential 8; OR (95%CI), odds ratio (95% confidence interval). [file 12889_2023_17456_MOESM1_ESM.docx]

**Cardiovascular health and life expectancy with and without cardiovascular disease in the middle-aged and elderly Chinese population**

**Supplementary Materials**

**Tables**

**Supplementary Table 1.** Baseline characteristics between excluded and included participants.

**Supplementary Table 2.** Definition and scoring approach for quantifying CVH of LE8.

**Supplementary Table 3.** Life expectancy and years lived with or without CVD at age 45 years by CVH Status of LE8, according to initial states of CVD.

**Supplementary Table 4.** Life expectancy and years lived with CVD at a given age from 45 years to 85 years by CVH Status of LE8 in men.

**Supplementary Table 5.** Life expectancy and years lived with CVD at a given age from 45 years to 85 years by CVH Status of LE8 in women.

**Supplementary Table 6.** Life expectancy and years lived with or without CVD at age 45 years by time-varying CVH Status of LE8.

**Supplementary Table 7.** Life expectancy and years lived with or without CVD at age 45 years by CVH Status of LE, excluding non-fatal cardiovascular events and deaths occurred in the first year.

**Supplementary Table 8.** Life expectancy and years lived with or without CVD at age 45 years by CVH Status of LE, excluding non-fatal cardiovascular events and deaths occurred in the first year.

**Figures**

**Supplementary Figure 1.** Flow chart of participants included and excluded in the analyses.

**Supplementary Figure 2.** Associations between CVH Status of component-specific LE8 and risk of incident CVD and all-cause mortality in men and women. Abbreviations: CVD, cardiovascular disease; CVH, cardiovascular health; LE8, Life’s Essential 8; OR (95%CI), odds ratio (95% confidence interval).

**Supplementary Table 1. Baseline characteristics between excluded and included participants.**

| **Characteristics** | **Included** | **Excluded** | ***P* value** |
| --- | --- | --- | --- |
| Participants, N (%) | 65587 | 35923 | <0.001 |
| Age at baseline, Mean (SD), in years | 57.0(8.7) | 40.6(40.9) | <0.001 |
| Men, N (%) | 53775(82.0) | 27326(76.1) | <0.001 |
| High school or above, N (%) | 8691(13.3) | 10920(30.4) | <0.001 |
| Personal monthly income ≥ 600 CNY, N (%) | 46780(71.3) | 26489(73.7) | <0.001 |
| Married, N (%) | 62365(95.1) | 29649(82.5) | <0.001 |
| Baseline history of CVD, N (%) | 3529(5.4) | 321(0.9) | <0.001 |

Values were represented by mean (SD) for continuous variable and frequency (%) for categorical variables. Differences of baseline characteristics between excluded and included participants were tested with t-test for continuous variables and Chi-square test for categorical variables. Abbreviations: CNY, Chinese yuan; SD, standard deviation.

**Supplementary Table 2. Definition and scoring approach for quantifying CVH of LE8.**

| **Domain** | **CVH metric** | **Method of measurement** | **Quantification of CVH metric** | |
| --- | --- | --- | --- | --- |
| Health behaviors | Diet | Measurement:  Self-reported intake of salt, fatty foods, and tea    Example tools for measurement:  What flavor do you prefer?  How often do you eat fatty foods?  How often do you drink tea? | Metric: Surrogate measurement of diet quality, calculated as the unweighted average of salt, fatty food, and tea scoring. | |
|  |  |  | Scoring: | |
|  |  |  | Points | Levels/Frequency |
|  |  |  | Salt intake | |
|  |  |  | 100 | < 6g/day |
|  |  |  | 50 | 6-12 g/day |
|  |  |  | 0 | > 12 g/day |
|  |  |  | Fatty food intake | |
|  |  |  | 100 | < 1 time/week |
|  |  |  | 50 | 1-3 times/week |
|  |  |  | 0 | >3 times/week |
|  |  |  | Tea consumption | |
|  |  |  | 100 | >3 times/week |
|  |  |  | 75 | 1-3 times/week |
|  |  |  | 50 | 1-3 times/month |
|  |  |  | 25 | < 1 time/month |
|  |  |  | 0 | Never |
|  | Physical activity | Measurements:  Self-reported minutes of physical activity per week.  Example tools for measurement:  How many times did you usually spend on physical activity  (Note: It took at least 20 minutes each time)? | Metric: Minutes of physical activity per week | |
|  |  |  | Scoring: | |
|  |  |  | Points | Minutes |
|  |  |  | 100 | ≥ 80 |
|  |  |  | 50 | 20-60 |
|  |  |  | 0 | < 20 |
|  | Tobacco/ Nicotine exposure | Measurements:  Self-reported use of cigarettes  Example tools for measurement:  Do you currently smoke cigarettes?  (Never smoker, former smoker, some days, every day) | Metric: Smoking | |
|  |  |  | Scoring: | |
|  |  |  | Points | Status |
|  |  |  | 100 | Never smoker |
|  |  |  | 50 | Former smoker, quit ≥ 1 year |
|  |  |  | 25 | Current smoker, < 1 cigarette/d |
|  |  |  | 0 | Current smoker, ≥ 1 cigarette/d |
|  | Sleep health | Measurements:  Self-reported average hours of sleep per night  Example tools for measurement:  On average, how many hours of sleep do you get per night? | Metric: Average hours of sleep per night | |
|  |  |  | Scoring: | |
|  |  |  | Points | Level |
|  |  |  | 100 | 7-< 9 |
|  |  |  | 90 | 9-< 10 |
|  |  |  | 70 | 6-< 7 |
|  |  |  | 40 | 5-< 6 or ≥ 10 |
|  |  |  | 20 | 4-< 5 |
|  |  |  | 0 | < 4 |
|  | Body mass index | Measurements:  Body weight (kg) divided by height squared(m²)  Example tools for measurement:  Objective measurement of height and weight | Metric: BMI (kg/m^2^) | |
|  |  |  | Scoring: | |
|  |  |  | Points | Level |
|  |  |  | 100 | < 23.0 |
|  |  |  | 70 | 23.0-24.9 |
|  |  |  | 30 | 25.0-29.9 |
|  |  |  | 15 | 30.0-34.9 |
|  |  |  | 0 | ≥ 35.0 |
| Health factors | Blood lipids | Measurements:  Plasma total and HDL cholesterol with the calculation of non-HDL cholesterol.  Example tools for measurement:  Fasting blood sample.  Non-HDL cholesterol unit conversion:  1 mg/dL=0.02586 mmol/L | Metric: Non-HDL cholesterol (mmol/L) | |
|  |  |  | Scoring: | |
|  |  |  | Points | Level |
|  |  |  | 100 | < 3.36 |
|  |  |  | 60 | 3.36-4.13 |
|  |  |  | 40 | 4.13-4.90 |
|  |  |  | 20 | 4.90-5.68 |
|  |  |  | 0 | ≥ 5.69 |
|  |  |  | If drug-treated level, subtract 20 points | |
|  | Blood glucose | Measurements:  FBG  Example tools for measurement:  Fasting blood sample.  FBG unit conversion:  1 mg/dL = 0.056 mmol/L | Metric: FBG (mmol/L) | |
|  |  |  | Scoring: | |
|  |  |  | Points | Level |
|  |  |  | 100 | No history of diabetes and FBG < 5.6 |
|  |  |  | 60 | No diabetes and FBG 5.6-6.9 |
|  |  |  | 40 | Diabetes with FBG < 8.6 |
|  |  |  | 30 | Diabetes with FBG 8.6-10.1 |
|  |  |  | 20 | Diabetes with FBG 10.2-11.6 |
|  |  |  | 10 | Diabetes with FBG 11.7-13.2 |
|  |  |  | 0 | Diabetes with FBG ≥ 13.3 |
| Health factors | Blood pressure | Measurements:  Appropriately measured systolic and diastolic BP  Example tools for measurement:  Corrected Mercury sphygmomanometer | Metric: Systolic and diastolic BPs (mmHg) | |
|  |  |  | Scoring: |  |
|  |  |  | Points | Level |
|  |  |  | 100 | <120/<80 (Optimal) |
|  |  |  | 75 | 120-129/<80 (Elevated) |
|  |  |  | 50 | 130-139 or 80-89 (Stage I hypertension) |
|  |  |  | 25 | 140-159 or 90-99 |
|  |  |  | 0 | ≥160 or ≥100 |
|  |  |  | Subtract 20 points if treated level | |

Abbreviations: BP, blood pressure; CVH, cardiovascular health; FBG, fasting blood glucose; HDL, high-density lipoprotein; LE8, Life’s Essential 8.

**Supplementary Table 3. Life expectancy and years lived with or without CVD at age 45 years by CVH Status of LE8, according to initial states of CVD.**

| **CVH status** | **Men** | | | | **Women** | | |
| --- | --- | --- | --- | --- | --- | --- | --- |
|  | **Life expectancy, years** | **Years lived without CVD,**  **years (%)** | **Years lived**  **with CVD, years (%)** | **Life expectancy, years** | | **Years lived without CVD,**  **years (%)** | **Years lived**  **with CVD, years (%)** |
| **Individuals without CVD** | | | | | | | |
| **Overall CVH** | | | | | | | |
| low CVH | 33.4 | 26.8(80.3) | 6.6(19.7) | 36.9 | | 30.3(82.3) | 6.5(17.7) |
| moderate CVH | 36.7 | 31.6(86.0) | 5.1(14.0) | 43.8 | | 38.6(88.1) | 5.2(11.9) |
| high CVH | 38.6 | 35.3(91.5) | 3.3(8.5) | 48.7 | | 44.5(91.4) | 4.2(8.6) |
| **CVH behaviors** | | | | | | | |
| low CVH | 35.5 | 30.1(84.8) | 5.4(15.2) | 40.4 | | 35.2(87.3) | 5.1(12.7) |
| moderate CVH | 36.6 | 31.4(85.9) | 5.2(14.1) | 44.2 | | 39.3(88.9) | 4.9(11.1) |
| high CVH | 38.0 | 33.2(87.4) | 4.8(12.6) | 46.2 | | 40.8(88.2) | 5.4(11.8) |
| **CVH factors** | | | | | | | |
| low CVH | 32.9 | 26.3(79.9) | 6.6(20.1) | 37.8 | | 31.3(82.6) | 6.6(17.4) |
| moderate CVH | 36.5 | 30.9(84.6) | 5.6(15.4) | 44.5 | | 39.2(88.2) | 5.3(11.8) |
| high CVH | 38.4 | 34.4(89.8) | 3.9(10.2) | 48.3 | | 44.1(91.2) | 4.3(8.8) |
| **Total** | 36.4 | 31.1(85.4) | 5.3(14.6) | 43.8 | | 38.8(88.5) | 5.1(11.5) |
| **Individuals with CVD** | | | | | | | |
| **Overall CVH** | | | | | | | |
| low CVH | 27.5 | - | 27.5(100.0) | 32.0 | | - | 32.0(100.0) |
| moderate CVH | 30.4 | - | 30.4(100.0) | 35.5 | | - | 35.5(100.0) |
| high CVH | 31.4 | - | 31.4(100.0) | 39.8 | | - | 39.8(100.0) |
| **CVH behaviors** | | | | | | | |
| low CVH | 29.8 | - | 29.8(100.0) | 35.6 | | - | 35.6(100.0) |
| moderate CVH | 30.2 | - | 30.2(100.0) | 34.5 | | - | 34.5(100.0) |
| high CVH | 30.6 | - | 30.6(100.0) | 39.0 | | - | 39.0(100.0) |
| **CVH factors** | | | | | | | |
| low CVH | 27.2 | - | 27.2(100.0) | 31.9 | | - | 31.9(100.0) |
| moderate CVH | 30.2 | - | 30.2(100.0) | 36.4 | | - | 36.4(100.0) |
| high CVH | 31.0 | - | 31.0(100.0) | 38.4 | | - | 38.4(100.0) |
| **Total** | 29.6 | - | 29.6(100.0) | 34.9 | | - | 34.9(100.0) |

Abbreviations: CVD, cardiovascular disease; CVH, cardiovascular health; LE8, Life’s Essential 8.

**Supplementary Table 4. Life expectancy and years lived with CVD at a given age from 45 years to 85 years by CVH Status of LE8 in men.**

| **Age, years** | **low CVH** | | **moderate CVH** | | **high CVH** | |
| --- | --- | --- | --- | --- | --- | --- |
|  | **Life expectancy, years** | **Years lived with CVD, years (%)** | **Life expectancy, years** | **Years lived with CVD, years (%)** | **Life expectancy, years** | **Years lived with CVD, years (%)** |
| 45.0 | 33.0 | 7.8(23.6) | 36.5 | 6.0(16.3) | 38.5 | 3.7(9.6) |
| 50.0 | 28.5 | 7.2(25.3) | 32.0 | 5.6(17.4) | 33.8 | 3.3(9.8) |
| 55.0 | 24.3 | 6.5(26.9) | 27.6 | 5.0(18.3) | 29.4 | 3.0(10.2) |
| 60.0 | 20.3 | 5.8(28.8) | 23.3 | 4.5(19.4) | 24.9 | 2.7(10.8) |
| 65.0 | 16.6 | 5.2(31.2) | 19.4 | 4.0(20.7) | 21.0 | 2.4(11.5) |
| 70.0 | 13.2 | 4.5(34.3) | 15.8 | 3.6(22.7) | 17.2 | 2.2(12.7) |
| 75.0 | 10.2 | 3.9(38.1) | 12.5 | 3.1(25.0) | 13.7 | 1.9(13.7) |
| 80.0 | 7.8 | 3.3(42.6) | 9.6 | 2.8(29.1) | 10.7 | 1.7(16.3) |
| 85.0 | 5.9 | 2.8(47.3) | 7.3 | 2.4(33.1) | 8.2 | 1.5(18.8) |

Proportion is computed by dividing years lived with CVD by total life expectancy at a given age from 45 years to 85 years. Abbreviations: CVD, cardiovascular disease; CVH, cardiovascular health; LE8, Life’s Essential 8.

**Supplementary Table 5. Life expectancy and years lived with CVD at a given age from 45 years to 85 years by CVH Status of LE8 in women.**

| **Age, years** | **low CVH** | | **moderate CVH** | | **high CVH** | |
| --- | --- | --- | --- | --- | --- | --- |
|  | **Life expectancy, years** | **Years lived with CVD, years (%)** | **Life expectancy, years** | **Years lived with CVD, years (%)** | **Life expectancy, years** | **Years lived with CVD, years (%)** |
| 45.0 | 36.6 | 7.8(21.3) | 43.6 | 6.0(13.7) | 48.6 | 4.5(9.3) |
| 50.0 | 32.2 | 7.4(23.1) | 38.8 | 6.0(15.5) | 43.9 | 4.5(10.2) |
| 55.0 | 27.7 | 7.0(25.1) | 34.2 | 5.6(16.3) | 39.2 | 4.5(11.4) |
| 60.0 | 23.5 | 6.4(27.2) | 29.7 | 5.2(17.5) | 34.5 | 4.1(11.9) |
| 65.0 | 19.6 | 5.9(30.1) | 25.4 | 4.9(19.2) | 30.0 | 3.8(12.8) |
| 70.0 | 16.0 | 5.3(33.2) | 21.4 | 4.5(21.2) | 25.7 | 3.6(14.1) |
| 75.0 | 12.7 | 4.7(36.9) | 17.6 | 4.1(23.5) | 21.8 | 3.4(15.6) |
| 80.0 | 9.9 | 4.0(40.9) | 14.1 | 3.7(26.1) | 17.9 | 3.0(17.0) |
| 85.0 | 7.5 | 3.4(45.2) | 11.0 | 3.3(29.8) | 14.5 | 2.7(18.5) |

Proportion is computed by dividing years lived with CVD by total life expectancy at a given age from 45 years to 85 years. Abbreviations: CVD, cardiovascular disease; CVH, cardiovascular health; LE8, Life’s Essential 8.

**Supplementary Table 6. Life expectancy and years lived with or without CVD at age 45 years by time-varying CVH Status of LE8.**

| **Time-varying**  **CVH status** | **Men** | | | **Women** | | |
| --- | --- | --- | --- | --- | --- | --- |
|  | **Life expectancy, years** | **Years lived without CVD,**  **years (%)** | **Years lived**  **with CVD, years (%)** | **Life expectancy, years** | **Years lived without CVD,**  **years (%)** | **Years lived**  **with CVD, years (%)** |
| **Overall** | | | | | | |
| low CVH | 33.5 | 25.8(77.0) | 7.7(23.0) | 38.1 | 31.2(81.7) | 7.0(18.3) |
| moderate CVH | 36.5 | 30.6(83.8) | 5.9(16.2) | 44.0 | 38.0(86.6) | 5.9(13.4) |
| high CVH | 37.6 | 33.9(90.0) | 3.8(10.0) | 48.7 | 44.1(90.6) | 4.6(9.4) |
| **Health behaviors** | | | | | | |
| low CVH | 35.3 | 28.9(82.0) | 6.4(18.0) | 41.9 | 35.9(85.7) | 6.0(14.3) |
| moderate CVH | 36.2 | 30.2(83.2) | 6.1(16.8) | 43.7 | 38.1(87.2) | 5.6(12.8) |
| high CVH | 38.2 | 32.0(83.8) | 6.2(16.2) | 46.1 | 39.2(85.0) | 6.9(15.0) |
| **Health factors** | | | | | | |
| low CVH | 33.6 | 25.2(75.2) | 8.3(24.8) | 39.6 | 32.1(80.9) | 7.6(19.1) |
| moderate CVH | 36.5 | 30.4(83.3) | 6.1(16.7) | 44.9 | 38.9(86.6) | 6.0(13.4) |
| high CVH | 37.6 | 33.5(89.0) | 4.1(11.0) | 45.9 | 41.7(90.9) | 4.2(9.1) |

Abbreviations: CVD, cardiovascular disease; CVH, cardiovascular health; LE8, Life’s Essential 8.

**Supplementary Table 7.** Life expectancy and years lived with or without CVD at age 45 years by CVH Status of LE, excluding non-fatal cardiovascular events and deaths occurred in the first year.

| **CVH status** | **Men** | | | **Women** | | |
| --- | --- | --- | --- | --- | --- | --- |
|  | **Life expectancy, years** | **Years lived without CVD, years (%)** | **Years lived with CVD, years (%)** | **Life expectancy, years** | **Years lived without CVD, years (%)** | **Years lived with CVD, years (%)** |
| **Overall CVH** | | | | | | |
| low CVH | 33.2 | 25.3(76.3) | 7.9(23.7) | 37.0 | 28.7(77.7) | 8.3(22.3) |
| moderate CVH | 36.4 | 30.3(83.3) | 6.1(16.7) | 44.2 | 37.6(85.0) | 6.6(15.0) |
| high CVH | 39.1 | 35.3(90.4) | 3.8(9.6) | 48.5 | 44.1(90.9) | 4.4(9.1) |
| **Health behaviors** | | | | | | |
| low CVH | 35.2 | 28.7(81.6) | 6.5(18.4) | 40.6 | 34.2(84.3) | 6.4(15.7) |
| moderate CVH | 36.3 | 30.0(82.6) | 6.3(17.4) | 44.0 | 38.3(87.0) | 5.7(13.0) |
| high CVH | 37.9 | 32.6(85.9) | 5.3(14.1) | 45.9 | 39.6(86.4) | 6.2(13.6) |
| **Health factors** | | | | | | |
| low CVH | 32.6 | 24.7(75.9) | 7.8(24.1) | 37.8 | 30.1(79.6) | 7.7(20.4) |
| moderate CVH | 36.2 | 29.5(81.3) | 6.8(18.7) | 44.2 | 38.2(86.3) | 6.1(13.7) |
| high CVH | 38.5 | 34.3(89.0) | 4.2(11.0) | 47.8 | 43.2(90.4) | 4.6(9.6) |

Abbreviations: CVD, cardiovascular disease; CVH, cardiovascular health; LE8, Life’s Essential 8.

**Supplementary Table 8.** Life expectancy and years lived with or without CVD at age 45 years by CVH Status of LE, excluding non-fatal cardiovascular events and deaths occurred in the first year.

| **CVH status** | **Men** | | | **Women** | | |
| --- | --- | --- | --- | --- | --- | --- |
|  | **Life expectancy, years** | **Years lived without CVD, years (%)** | **Years lived with CVD, years (%)** | **Life expectancy, years** | **Years lived without CVD, years (%)** | **Years lived with CVD, years (%)** |
| **Overall CVH** | | | | | | |
| low CVH | 33.2 | 25.3(76.3) | 7.9(23.7) | 37.0 | 28.7(77.7) | 8.3(22.3) |
| moderate CVH | 36.4 | 30.3(83.3) | 6.1(16.7) | 44.2 | 37.6(85.0) | 6.6(15.0) |
| high CVH | 39.1 | 35.3(90.4) | 3.8(9.6) | 48.5 | 44.1(90.9) | 4.4(9.1) |
| **Health behaviors** | | | | | | |
| low CVH | 35.2 | 28.7(81.6) | 6.5(18.4) | 40.6 | 34.2(84.3) | 6.4(15.7) |
| moderate CVH | 36.3 | 30.0(82.6) | 6.3(17.4) | 44.0 | 38.3(87.0) | 5.7(13.0) |
| high CVH | 37.9 | 32.6(85.9) | 5.3(14.1) | 45.9 | 39.6(86.4) | 6.2(13.6) |
| **Health factors** | | | | | | |
| low CVH | 32.6 | 24.7(75.9) | 7.8(24.1) | 37.8 | 30.1(79.6) | 7.7(20.4) |
| moderate CVH | 36.2 | 29.5(81.3) | 6.8(18.7) | 44.2 | 38.2(86.3) | 6.1(13.7) |
| high CVH | 38.5 | 34.3(89.0) | 4.2(11.0) | 47.8 | 43.2(90.4) | 4.6(9.6) |

Abbreviations: CVD, cardiovascular disease; CVH, cardiovascular health; LE8, Life’s Essential 8.

**
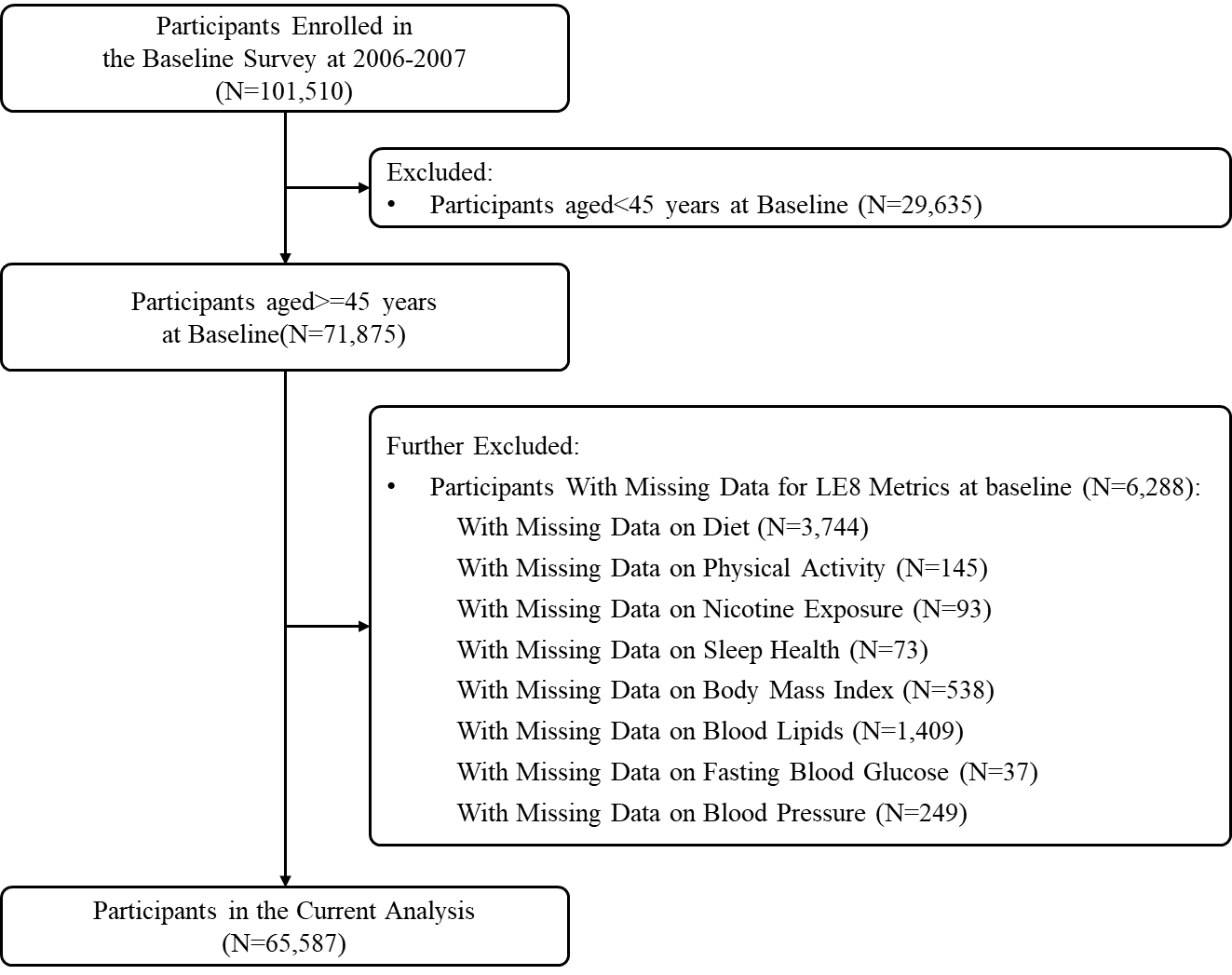
**

**Supplementary Figure 1.** Flow chart of participants included and excluded in the analyses.


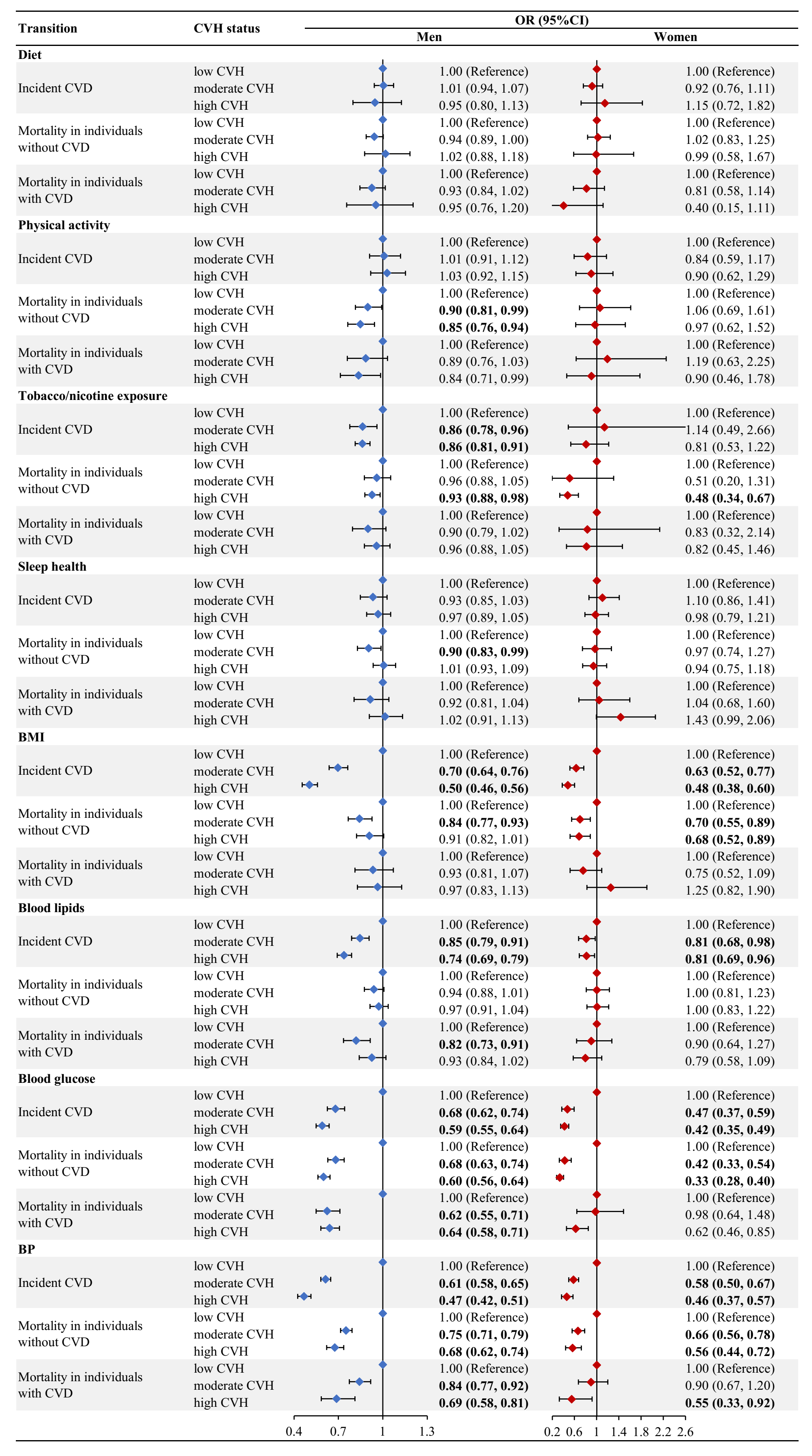


**Supplementary Figure 2.** Associations between CVH Status of component-specific LE8 and risk of incident CVD and all-cause mortality in men and women. Abbreviations: CVD, cardiovascular disease; CVH, cardiovascular health; LE8, Life’s Essential 8; OR (95%CI), odds ratio (95% confidence interval).
